# Food and Housing Insecurity, Resource Allocation, and Follow-up in a Pediatric Emergency Department

**DOI:** 10.5811/westjem.19435

**Published:** 2025-01-15

**Authors:** Raymen R. Assaf, Chloe Knudsen-Robbins, Theodore Heyming, Kellie Bacon, Shelby K. Shelton, Bharath Chakravarthy, Soheil Saadat, Jason A. Douglas, Victor Cisneros

**Affiliations:** * Children’s Hospital of Orange County, Orange, California; † University of California Irvine, School of Medicine, Department of Pediatrics, Irvine, California; ‡ University of Cincinnati College of Medicine, Department of Emergency Medicine, Cincinnati, Ohio; § University of California at Irvine, Department of Emergency Medicine, Orange, California; ∥ University of California Irvine, Department of Health, Society, & Behavior, Irvine, California; ¶ Eisenhower Health, Department of Emergency Medicine, Rancho Mirage, California; # University of California Riverside, School of Medicine, Department of Emergency Medicine, Riverside, California

## Abstract

**Introduction:**

Food and housing insecurity in childhood is troublingly widespread. Emergency departments (ED) are well positioned to identify and support food- and housing-insecure children and their families. However, there is no consensus regarding the most efficient screening tools or most effective interventions for ED use.

**Objective:**

In this cross-sectional study we aimed to investigate the implementation of a food/ housing insecurity screening tool and resource referral uptake in a pediatric ED.

**Methods:**

During the study period (March 1–December 9, 2021), there were 67,297 ED visits at the study institution, which is a freestanding children’s hospital. Caregivers of patients presenting to the ED were approached for participation in the study; 1,908 families participated (2.8% of all ED visits during the study period) and were screened for food and housing insecurity. Caregiver surveys included demographic, food and housing insecurity, caregiver/patient health status, and healthcare utilization questions. Caregivers who screened positive for food and/or housing insecurity received printed materials with food and/or housing resources. We analyzed data using descriptive statistics, one-way analysis of variance, and the Pearson chi-squared test.

**Results:**

A total of 1,908 caregivers were surveyed: 416 (21.8%) screened positive for food and/or housing insecurity. Of those who screened positive, 147/416 completed follow-up surveys. On follow-up, 44 (30.0%) no longer screened positive for food and/or housing insecurity, while 15 (10.2%) reported using at least one resource referral. The most frequently reported referral utilization barrier was loss or reported non-receipt of the referral.

**Conclusion:**

This study demonstrates high food- and housing-insecurity rates among families presenting to a pediatric ED, emphasizing the urgency and necessity of screening and intervening in this environment. The food and housing insecurity change between baseline and follow-up reported here and the overall low resource uptake highlights challenges with ED-based screening and intervention efficacy.

Population Health Research Capsule What do we already know about this issue?

*Food and housing insecurity interventions are increasing in the pediatric emergency department (ED) yet lack a standard approach to optimizing resource utilization.*

What was the research question?

*What are barriers to uptake of food and housing insecurity community-resource referrals in a pediatric ED?*

What was the major finding of the study?

*On follow-up, only 10% of participating families reported using at least one resource referral.*

How does this improve population health?

*This study identifies multiple barriers to community resource use and follow-up among families participating in a passive referral approach in a pediatric ED.*

## INTRODUCTION

One in six of all children in the United States (US) are food insecure, while one in 18 under the age of six are unhoused.^1^ In 2017, over 1.5 million children enrolled in public schools were unhoused.^2^ ^,^ ^3^ Beyond a statistical representation of societal shortcomings of meeting the basic needs of children, these figures are distressing as food and housing insecurity has repeatedly been shown to be associated with adverse mental, physical, and developmental health outcomes.^1^ ^,^ ^4^ ^–^ ^12^ Food and housing insecurity disproportionately burdens underserved communities of color, particularly those in which more than 20% of residents live in poverty, and downstream health disparities are common.^13^

Since 2015 the American Academy of Pediatrics has advocated for the screening of food insecurity during well-child visits, and this approach has now expanded to a variety of healthcare settings.^14^ The emergency department (ED) is particularly well positioned to assess for health-related social needs (HRSN) and to potentially intervene. Over 15% of all US children visit the ED each year, many with barriers to routine preventive care, and food/housing insecurity has been shown to be associated with increased ED use.^15^ ^–^ ^18^ Several studies have demonstrated the feasibility of various screening methods and resource referral for food and housing insecurity in the ED.^19^ ^–^ ^25^ However, there is no current consensus regarding the most effective techniques for reliable, widespread screening in the ED or recommendations for optimizing caregiver resource utilization. In this study we aimed to investigate the implementation of a food and housing insecurity screening tool and resource referral uptake in a pediatric ED.

## METHODS

This cross-sectional study included patients presenting to the ED of a freestanding children’s hospital with a Level II trauma center between March 1–December 9, 2021. This institution, located in a suburban community in the Southwestern US, has an annual ED census of approximately 100,000 visits per year; 67,297 visits occurred during the study period. In the study county, approximately 24% of households report a household income of under $50,000/year, 25% report $50,000–100,000, 31% report $100,000–$200,000, and 19% report over $200,000. An estimated 11% of children live below the poverty line.^26^ This study was approved by the study institution’s institutional review board (IRB# 200326).

Using a convenience sample of adult caregivers of patients <18, trained research assistants (RA) approached prospective participants during triage, described the study, invited them to participate, and obtained verbal consent from those who agreed. The RAs approached eligible patient caregivers during the hours of 8 am – 5 pm Monday through Friday during the study period. The RAs administered surveys via REDCap (Research Electronic Data Capture hosted at UC Irvine Emergency Department) on electronic tablets, in which participants directly entered their responses. Surveys were available in English and Spanish. The baseline survey included an expanded demographics section followed by 16 questions regarding food insecurity, access to food, housing insecurity, neighborhood safety, caregiver self-reported health, caregiver-reported patient health, and healthcare utilization (Supplementary Materials Appendix A). We also garnered caregiver self-reported race/ethnicity as well as insurance status from patient registration data. Surveys were developed by RA, VC, and JD, authors with expertise in public health.

We assessed and defined food insecurity based on two previously validated screening questions: “Within the past 12 months, I worried whether my food would run out before I got money to buy more”; and “Within the past 12 months, the food I bought just didn’t last and I didn’t have money to get more.”^27^ Affirmative responses to either or both questions was considered a positive screen. We assessed housing instability on an affirmative response either to 1) “In the past 12 months, have you had trouble paying your rent/mortgage/ utility bills,” or 2) a response of “Stay at a friend’s home” or “I do not live in stable housing” to the survey question “In the past 12 months, have you been living in stable housing that you…” This definition is consistent with prior studies, although historically housing instability has been defined by various criteria in federal bodies and scientific literature, rendering it more difficult to consistently measure than food insecurity.^4^

All caregivers who completed the survey received curated printed materials with current local food and/or housing resources. The RAs provided these resources immediately after the participants completed the survey. Direct communication between research personnel and community resources about individual-level need (eg, warm hand-offs) were not part of the study methods. The study institution’s social work team worked with authors RA and VC to develop documents containing an extensive list of vetted local community resources. Additionally, those who screened positive for food or housing insecurity were contacted by RAs three weeks and six weeks after the index ED visit to conduct follow-up surveys. The RAs conducted follow-up surveys via telephone and attempted to contact families up to three times. Follow-up surveys included questions regarding use of provided resources, barriers to use, and food/housing insecurity in the prior three weeks (Supplementary Materials Appendix B). Follow-up status (food insecure, housing insecure, or both food and housing insecure) was recorded based on final responses (ie, at three weeks if caregivers didn’t respond to the six-week survey or six weeks if they responded to both surveys).

### Statistical Analysis

Data was screened and cleaned prior to analyses by PKP. Descriptive statistics were used to analyze demographic, healthcare utilization, and clinical characteristics. We analyzed patient age and ED length of stay (both continuous variables) using the Fisher *t*-test or Welch one-way analysis of variance. All other variables (categorical) were analyzed using the Pearson chi-squared test with Monte Carlo simulation and standardized residuals (z) to interpret significant associations.

## RESULTS

A total of 2,144 adult caregivers participated in the survey. Initial food/housing insecurity status was indeterminate for 236 patients as their caregivers did not respond to the food/housing questions described above and, thus, this group was excluded from data analysis. Of the remaining 1,908 respondents (2.8% of total ED visits during the study period), a total of 416 (21.8%) screened positive for food and/or housing insecurity. Additionally, 164 caregivers (8.6%) screened positive for food and housing insecurity, 95 (4.98%) for solely food insecurity, and 157 (8.2%) for solely housing insecurity.

### Initial Survey

#### Demographics

The mean age for all patients whose caregiver completed a survey was 6.68 ± 5.26 years. On average, patients with food or housing insecurity (7.42 ± 5.40 years), food and housing insecurity (7.95 ± 5.44 years) were older than those without (6.41 ± 5.18 years; *P* < 0.001). Slightly more than half of all patients were male (52.8%); there was no significant difference with respect to sex among patients with and without food and/or housing insecurity; *P* = 0.43. Among those surveyed, 64.1% were Hispanic, 20.4% White non-Hispanic, 7.3% Asian, and 2.5% Black. Just over 75% of caregivers who screened positive for food or housing security were Hispanic, 11.5% were White non-Hispanic, 4.4% were Asian, and 2.0% were Black. Caregivers who were both food and housing secure were more likely to report White non-Hispanic race and ethnicity (*z* = 2.9, *P* < 0.001). Over two-thirds, 67.5%, of patients had public health insurance; caregivers who were food and/or housing insecure were more likely to have public health insurance than private health insurance (*z* = 4.22, *z* = 4.41, *P* < 0.001). Complete demographics stratified by total population, and those with and without food and/or housing insecurity are included in Table 1.

**Table 1.** Initial survey: demographics and clinical characteristics as stratified by food and housing insecurity status.

		Baseline status (N = 1,908)			
Characteristics	Total population (N = 1,908)	Food or housing insecure (n = 252)	Both food and housing insecure (n = 164)	Both food and housing secure (n = 1,492)	*P*-value
*Demographic*					
Patient age, mean years (SD)	6.68 (5.26)	7.42 (5.40)	7.95 (5.44)	6.41 (5.18)	<0.001
Patient male sex, n (%)	1,008 (52.8%)	124 (49.2%)	90 (54.9%)	794 (53.2%)	0.43
Patient ethnicity and race					<0.001
White, non-Hispanic, n (%)	389 (20.4%)	29 (11.5%)	19 (11.6%)	341 (22.9%)	
Hispanic, n (%)	1,223 (64.1%)	191 (75.8%)	128 (78.0%)	904 (60.6%)	
Black, n (%)	47 (2.5%)	5 (2.0%)	4 (2.4%)	38 (2.5%)	
Asian, n (%)	139 (7.3%)	11 (4.4%)	4 (2.4%)	124 (8.3%)	
Other (multiethnic, multiracial, etc), n (%)	110 (5.8%)	16 (6.3%)	9 (5.5%)	85 (5.7%)	
Missing, n (%)	0 (0.0%)	0 (0.0%)	0 (0.0%)	0 (0.0%)	
Patient health insurance					<0.001
Public, n (%)	1,287 (67.5%)	225 (89.3%)	157 (95.7%)	905 (60.7%)	
Private, n (%)	597 (31.3%)	26 (10.3%)	6 (3.7%)	565 (37.9%)	
Military, n (%)	20 (1.0%)	0 (0.0%)	0 (0.0%)	20 (1.3%)	
Self-pay, n (%)	4 (0.2%)	1 (0.4%)	1 (0.6%)	2 (0.1%)	
Language spoken at home					<0.001
English, n (%)	1,380 (72.3%)	150 (59.3%)	95 (57.9%)	1,135 (76.1%)	
Spanish, n (%)	451 (23.6%)	94 (37.3%)	64 (39.0%)	293 (19.6%)	
Vietnamese, n (%)	25 (1.3%)	4 (1.6%)	2 (1.2%)	19 (1.3%)	
Other, n (%)	52 (2.7%)	4 (1.6%)	3 (1.8%)	45 (3.0%)	
Household income					<0.001
<$20,000, n (%)	325 (17.0%)	80 (31.7%)	58 (35.4%)	187 (12.5%)	
$20,000 – $39,999, n (%)	427 (22.4%)	73 (29.0%)	57 (34.8%)	297 (19.9%)	
$40,000 – $59,999, n (%)	253 (13.3%)	38 (15.1%)	20 (12.2%)	195 (13.1%)	
$60,000 – $79,999, n (%)	131 (6.9%)	14 (5.5%)	2 (1.2%)	115 (7.7%)	
$80,000 – $99,999, n (%)	77 (4.0%)	4 (1.6%)	3 (1.8%)	70 (4.7%)	
≥$100,000, n (%)	357 (18.7%)	7 (2.8%)	1 (0.6%)	349 (23.4%)	
Missing or prefer not to answer, n (%)	338 (17.7%)	36 (14.3%)	23 (14.0%)	279 (18.7%)	
Respondent’s highest education level					<0.001
Less than high school, n (%)	68 (3.6%)	16 (6.3%)	7 (4.3%)	45 (3.0%)	
Some high school, n (%)	162 (8.5%)	42 (16.7%)	31 (18.9%)	89 (6.0%)	
High school diploma or GED, n (%)	499 (26.3%)	71 (28.2%)	54 (32.9%)	374 (25.1%)	
Some college, n (%)	594 (31.1%)	77 (30.5%)	48 (29.3%)	469 (31.4%)	
College degree, n (%)	542 (28.4%)	37 (14.7%)	21 (12.8%)	484 (32.4%)	
Missing or prefer not to answer, n (%)	43 (2.3%)	9 (3.6%)	3 (1.8%)	31 (2.1%)	
Number of times moved during past 12 months					<0.001
0, n (%)	1,294 (67.8%)	141 (56%)	88 (53.6%)	1,065 (71.4%)	
1, n (%)	274 (14.4%)	55 (21.8%)	39 (23.8%)	180 (12.1%)	
2, n (%)	50 (2.6%)	20 (7.9%)	11 (6.7%)	19 (1.3%)	
≥3, n (%)	20 (1.0%)	7 (2.8%)	8 (4.9%)	5 (0.3%)	
Missing or prefer not to answer, n (%)	270 (14.2%)	29 (11.5%)	18 (11.0%)	223 (14.9%)	
Respondent’s perception of neighborhood safety: “Do you feel safe in your neighborhood?”					<0.001
Always, n (%)	1,391 (72.9%)	150 (59.5%)	69 (42.1%)	1,172 (78.6%)	
Usually, n (%)	377 (19.8%)	68 (27.0%)	53 (32.3%)	256 (17.2%)	
Sometimes, n (%)	85 (4.5%)	24 (9.5%)	32 (19.5%)	29 (1.9%)	
Never, n (%)	22 (1.2%)	3 (1.2%)	5 (3.0%)	14 (0.9%)	
Missing, n (%)	33 (1.7%)	7 (2.8%)	5 (3.0%)	21 (1.4%)	
Respondent’s concern for patient’s safety in neighborhood: “Are you concerned about your child’s safety in your neighborhood?”					<0.001
Always, n (%)	81 (4.2%)	16 (6.3%)	9 (5.5%)	56 (3.7%)	
Usually, n (%)	49 (2.6%)	9 (3.6%)	8 (4.9%)	32 (2.1%)	
Sometimes, n (%)	273 (14.3%)	57 (22.6%)	57 (34.8%)	159 (10.7%)	
Never, n (%)	1,453 (76.2%)	157 (62.3%)	82 (50.0%)	1,214 (81.4%)	
Missing, n (%)	52 (2.7%)	13 (5.2%)	8 (4.9%)	31 (2.1%)	
Respondent’s perception of patient health					<0.001
Excellent, n (%)	674 (35.3%)	60 (23.8%)	34 (20.7%)	580 (38.9%)	
Very good, n (%)	654 (34.3%)	97 (38.5%)	50 (30.5%)	507 (34.0%)	
Good, n (%)	442 (23.2%)	68 (27.0%)	55 (33.5%)	319 (21.4%)	
Fair, n (%)	110 (5.8%)	24 (9.5%)	22 (13.4%)	64 (4.3%)	
Poor, n (%)	22 (1.2%)	2 (0.8%)	3 (1.8%)	17 (1.1%)	
Missing, n (%)	6 (0.3%)	1 (0.4%)	0 (0.0%)	5 (0.3%)	
Respondent’s perception of own health					<0.001
Excellent, n (%)	479 (25.1%)	41 (16.3%)	26 (15.9%)	412 (27.6%)	
Very good, n (%)	676 (35.4%)	79 (31.3%)	32 (19.5%)	565 (37.9%)	
Good, n (%)	594 (31.1%)	91 (36.1%)	64 (39.0%)	439 (29.4%)	
Fair, n (%)	148 (7.8%)	39 (15.5%)	37 (22.6%)	72 (4.8%)	
Poor, n (%)	10 (0.5%)	2 (0.8%)	5 (3.0%)	3 (0.2%)	
Missing, n (%)	1 (0.1%)	0 (0.0%)	0 (0.0%)	1 (0.1%)	
*Clinical*					
Emergency severity index					0.05^*^
Level 5, n (%)	33 (1.7%)	0 (0.0%)	5 (3.0%)	28 (1.9%)	
Level 4, n (%)	351 (18.4%)	50 (19.8%)	34 (20.7%)	267 (17.9%)	
Level 3, n (%)	1,243 (65.1%)	172 (68.3%)	109 (66.5%)	962 (64.5%)	
Level 2, n (%)	281 (14.7%)	30 (11.9%)	16 (9.8%)	235 (15.8%)	
ED disposition					0.27
Discharged, n (%)	1,544 (80.9%)	206 (81.7%)	138 (84.1%)	1,200 (80.4%)	
Admitted, n (%)	354 (18.6%)	43 (17.1%)	24 (14.6%)	287 (19.2%)	
Transferred, n (%)	9 (0.5%)	3 (1.1%)	2 (1.2%)	4 (0.3%)	
Left against medical advice, n (%)	1 (0.1%)	0 (0.0%)	0 (0.0%)	1 (0.1%)	
ED length of stay, mean hours (SD)	4.52 (3.19)	4.92 (2.80)	4.82 (2.87)	4.85 (3.45)	0.95
Number of ED visits during past 12 months					<0.001
0, n (%)	1,304 (68.3%)	160 (63.5%)	94 (57.3%)	1,050 (70.4%)	
1, n (%)	374 (19.6%)	49 (19.4%)	49 (29.9%)	276 (18.5%)	
2 or more, n (%)	230 (12.1%)	43 (17.1%)	21 (12.8%)	166 (11.1%)	
A doctor has stated that patient has (check all that apply):	1,479 (77.5%)	171 (67.9%)	103 (62.8%)	1,205 (80.7%)	
None of those listed					
Asthma, n (%)	224 (11.7%)	39 (15.5%)	19 (11.6%)	166 (11.1%)	0.05^*^
Missing, n (%)	31 (1.6%)	17 (6.7%)	14 (8.5%)	0 (0.0%)	
Obesity, n (%)	61 (3.2%)	9 (3.6%)	12 (7.3%)	40 (2.7%)	0.003
Missing, n (%)	34 (1.8%)	20 (7.9%)	14 (8.5%)	0 (0.0%)	
Diabetes, n (%)	25 (1.3%)	4 (1.6%)	2 (1.2%)	19 (1.3%)	0.93
Missing, n (%)	34 (1.8%)	20 (7.9%)	14 (8.5%)	0 (0.0%)	
Anxiety, n (%)	94 (4.9%)	13 (5.2%)	18 (11.0%)	63 (4.2%)	<0.001
Missing, n (%)	35 (1.8%)	21 (8.3%)	14 (8.5%)	0 (0.0%)	
Emotional challenges, n (%)	87 (4.6%)	14 (5.6%)	17 (10.4%)	56 (3.8%)	<0.001
Missing, n (%)	35 (1.8%)	21 (4.4%)	14 (8.5%)	0 (0.0%)	
Behavioral difficulties, n (%)	50 (2.6%)	11 (4.4%)	9 (5.5%)	30 (2.0%)	0.002^*^
Missing, n (%)	36 (1.9%)	21 (8.3%)	15 (9.1%)	0 (0.0%)	

* *Note:* Despite this significant *P*-value, none of the *z*’s were ≥2.58; Type 1 error possible.

*ED*, emergency department; *GED*, General Educational Development.

#### Neighborhood safety

Of all caregivers surveyed, 72.9% reported always feeling safe in their neighborhood and 76.2% reported never being concerned about the patient’s safety in their neighborhood. Caregivers screening positive for both food and housing insecurity were less likely to report always feeling safe in their neighborhood (*z* = −4.5) and more likely to report sometimes being concerned about the patient’s safety in their neighborhood (*z* = −4.5, *z* = 7.1, *P* < 0.001).

#### Health status and healthcare utilization

Only 35.3% of caregivers rated the patient’s health as excellent, while even fewer, 25.1%, rated their own health as excellent. Those screening positive for housing and/or food insecurity were less likely to rate the patient’s health as excellent (*z* = −3.1, *z* = −3.2, *P* < 0.001) as well as their own health as excellent or very good (*z* = −2.8, *z* = −3.4, *P* < 0.001). Caregivers who screened positive for both housing and food insecurity were more likely to report that at some time a physician told them the patient was obese (*z* = 3.22), had anxiety (*z* = 3.82), or had emotional challenges (*z* = 3.80, *P* < 0.001).

In our study, 68.3% of caregivers reported the patient had not visited the ED in the previous year, 19.6% reported a single visit, and 12.1% reported two or more visits during the same time frame. Caregivers screening positive for both food and housing insecurity were more likely to report visiting the ED at least once (*z* = 3.0, *P* < 0.001).

#### ED visit characteristics

The majority (65.1%) of patients were triaged to Emergency Severity Index (ESI) level 3 and discharged home from the index ED visit (80.9%), while the mean length of stay in the ED was 4.5 ± 3.19 hours. There was no significant difference in ED disposition or length of stay among caregivers reporting food and/or housing insecurity compared to those who were food and housing secure.

#### Moves in the preceding year

Baseline survey results indicated that 20 caregivers (1.0% of the sample) reported moving three or more times in the previous 12 months. Of those, none screened positive for solely food insecurity, 35% screened positive for solely housing insecurity, 40% screened positive for both food and housing insecurity, and 25% did not screen positive for food or housing insecurity.

### Follow-up

Of the 416 families screening positive for food or housing insecurity, contact was successfully made with 147 (35.3%) caregivers at three weeks, and of those, 70 (47.6%) responded to surveys at six weeks post-ED visit.

#### Food/housing insecurity status

Of the 147 caregivers who participated in follow-up, 25 were solely food insecure at the index ED visit. Of those, seven (28%) continued to report food insecurity at the time of follow-up, two (8%) reported solely housing instability without food insecurity, three (12%) reported both food and housing insecurity, and 12 (48%) no longer screened positive for either food or housing insecurity. Of the 60 caregivers who screened positive for solely housing insecurity at the index ED visit and participated in follow-up, 19 (31.7%) continued to report housing insecurity at the time of follow-up, three (5%) reported new food insecurity only, 11 (18.3%) reported both food and housing insecurity, and 26 (43%) no longer screened positive for either food or housing insecurity.

Of the 62 caregivers who screened positive for both food and housing insecurity at the index ED visit and participated in follow-up, 31 (50%) continued to report both food and housing insecurity, 15 (24.2%) reported food insecurity only, 10 (16.1%) reported housing insecurity only, and six (9.7%) no longer screened positive for food or housing insecurity. Follow-up status was unknown due to missing data for two families. (One reported food insecurity, and the other reported housing instability at the index ED visit.)

#### Transitions from positive food and/or housing insecurity screening to negative screening

Of the 147 caregivers who reported food and/or housing insecurity at the index ED visit and participated in follow-up, 44 (29.9%) no longer screened positive for either food or housing insecurity at follow-up. Families of those initially screening positive who subsequently did not screen positive appeared generally similar with respect to demographics, neighborhood safety, health status/healthcare utilization, and ED visit characteristics (Table 2). Younger patient age was associated with a transition from a positive to negative screen (*P* = 0.02). Table 2 includes comparisons of all collected variables for these two groups. Given the relatively low number of families that followed up and reported resource use, it was not possible to determine whether there was any association between referral use and transition from positive to negative screens.

**Table 2.** Follow-up survey: demographics and clinical characteristics as stratified by food/housing insecurity status.

	Follow-up status^*^		
Characteristics	Both food and housing secure (n = 44)	Food and/or housing insecure (n = 101)	*P*-value
*Demographic*			
Patient age, mean years (SD)	5.87 (5.14)	8.22 (5.37)	0.02
Patient male sex, n (%)	27 (61.4%)	48 (47.5%)	0.15
Patient ethnicity and race			0.68
Hispanic, n (%)	30 (68.2%)	80 (79.2%)	
Non-Hispanic White, n (%)	8 (18.2%)	11 (10.9%)	
Black, n (%)	1 (2.3%)	2 (2.0%)	
Asian, n (%)	3 (6.8%)	4 (4.0%)	
Other, n (%)	2 (4.6%)	4 (4.0%)	
Patient health insurance			0.02^**^
Public, n (%)	37 (84.1%)	97 (96.0%)	
Private, n (%)	7 (15.9%)	4 (4.0%)	
Language spoken at home			0.72
English, n (%)	31 (70.5%)	77 (76.2%)	
Spanish, n (%)	11 (25.0%)	21 (20.8%)	
Other, n (%)	2 (4.6%)	3 (3.0%)	
Household income			0.01^**^
<$20,000, n (%)	12 (27.3%)	36 (35.6%)	
$20,000 – $39,999, n (%)	13 (29.6%)	40 (39.6%)	
$40,000 – $59,999, n (%)	7 (15.9%)	12 (11.9%)	
$60,000 – $79,999, n (%)	6 (13.6%)	4 (4.0%)	
$80,000 – $99,999, n (%)	2 (4.6%)	0 (0%)	
≥$100,000, n (%)	2 (4.6%)	0 (0%)	
Missing, n (%)	2 (4.6%)	9 (8.9%)	
Respondent’s highest education level			0.47
Less than high school, n (%)	2 (4.6%)	6 (5.9%)	
Some high school, n (%)	7 (15.9%)	17 (16.8%)	
High school diploma or GED, n (%)	10 (22.7%)	32 (31.7%)	
Some college, n (%)	14 (31.8%)	33 (32.7%)	
College degree, n (%)	11 (25.0%)	12 (11.9%)	
Missing, n (%)	0 (0%)	1 (1.0%)	
Number of times moved during past 12 months			0.76
0, n (%)	26 (59.1%)	59 (58.4%)	
1, n (%)	11 (25.0%)	25 (24.8%)	
2, n (%)	2 (4.6%)	5 (5.0%)	
≥3, n (%)	1 (2.3%)	7 (6.9%)	
Missing, n (%)	4 (9.1%)	5 (5.0%)	
Respondent’s perception of neighborhood safety, n (%)			0.79
Always	26 (59.1%)	55 (54.5%)	
Usually	12 (27.3%)	34 (33.7%)	
Sometimes	3 (6.8%)	9 (8.9%)	
Never	0 (0.0%)	1 (1.0%)	
Missing, n (%)	3 (6.8%)	2 (2.0%)	
Respondent’s concern for patient’s safety in neighborhood, n (%)			0.47
Always	3 (6.8%)	6 (5.9%)	
Usually	3 (6.8%)	5 (5.0%)	
Sometimes	8 (18.2%)	31 (30.7%)	
Never	27 (61.4%)	52 (51.5%)	
Missing, n (%)	3 (6.8%)	7 (6.9%)	
Respondent’s perception of patient health, mean (SD)			0.34
Excellent	8 (18.2%)	23 (22.8%)	
Very good	18 (40.9%)	39 (38.6%)	
Fair	11 (25%)	32 (31.7%)	
Poor	7 (15.9%)	7 (6.9%)	
Missing, n (%)	0 (0.0%)	0 (0.0%)	
	0 (0.0%)	0 (0.0%)	
Respondent’s perception of own health, mean (SD)			0.76
Excellent, n (%)	6 (13.6%)	13 (12.9%)	
Very good, n (%)	13 (29.5%)	31 (30.7%)	
Good, n (%)	19 (43.2%)	41 (40.6%)	
Fair, n (%)	6 (13.6%)	12 (11.9%)	
Poor, n (%)	0 (0.0%)	4 (4.0%)	
Missing, n (%)	0 (0.0%)	0 (0.0%)	
*Clinical*			
Emergency severity index			0.76
Level 5, n (%)	1 (2.3%)	1 (1.0%)	
Level 4, n (%)	5 (11.4%)	18 (17.8%)	
Level 3, n (%)	32 (72.7%)	70 (69.3%)	
Level 2, n (%)	6 (13.6%)	12 (11.9%)	
ED disposition			1.00
Discharged, n (%)	37 (84.1%)	84 (83.2%)	
Admitted, n (%)	6 (13.6%)	15 (14.9%)	
Transferred, n (%)	1 (2.3%)	2 (2.0%)	
ED length of stay, mean hours (SD)	5.50 (3.57)	4.58 (2.33)	0.13
Number of ED visits during past 12 months, n (%)			<0.001^**^
0, n (%)	25 (56.8%)	60 (59.4%)	
1, n (%)	3 (6.8%)	28 (2.8%)	
2 or more, n (%)	16 (36.4%)	13 (12.9%)	
A doctor has stated that patient has: none of those listed, n (%)	29 (65.9%)	69 (68.3%)	0.99
Asthma, n (%)	9 (20.5%)	12 (11.9%)	0.20
Obesity, n (%)	3 (6.8%)	7 (6.9%)	1.00
Diabetes, n (%)	1 (2.3%)	0 (0%)	0.30
Anxiety, n (%)	4 (9.1%)	11 (10.9%)	1.00
Emotional challenges, n (%)	3 (6.8%)	13 (12.9%)	0.39
Behavioral difficulties, n (%)	3 (6.8%)	7 (6.9%)	1.00

* Follow-up status unknown due to missing data for two families (one food insecure and one housing insecure at baseline).

** Despite this significant *P*-value, none of the *z*’s were ≥2.58; Type 1 error possible.

*ED*, emergency department; *GED*, General Educational Development.

#### Reported barriers to resource use

Of the 147 caregivers who participated in follow-up, only 15 (10.2%) reported using at least one of the resource referrals. The most frequently reported barrier for those reporting a barrier to resource use was losing or not receiving the referral (41.7%). Other common reasons included not having time (15.2%) and resources not fitting their needs (10.6%). The Figure demonstrates caregiver-reported barriers to referral use.

**Figure.**
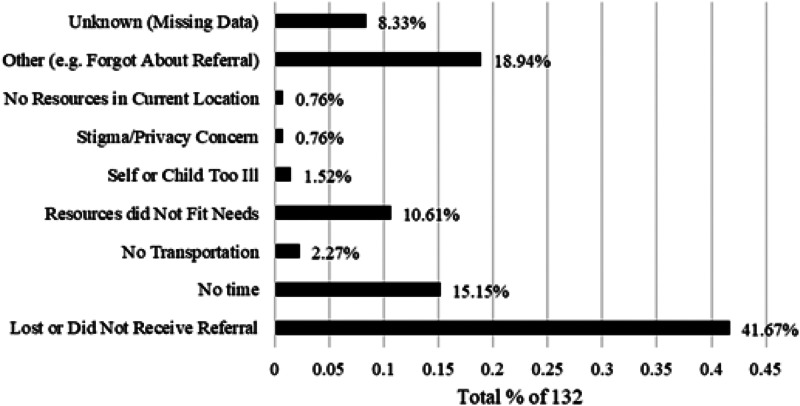
Reported barriers to referral use.

#### Demographics of those with and without follow-up

Patients whose caregivers participated in follow-up had largely similar demographics to those who did not, except for language spoken at home. Spanish-speaking caregivers were less well represented among those with follow-up (*z* = −3.06, *P* < 0.001). Supplementary Table 1 demonstrates demographics, neighborhood safety, health status, healthcare utilization, and ED visit characteristics of those with and without follow-up.

## DISCUSSION

This study demonstrated high levels of social need in patients presenting to a pediatric ED. Over one in five patients screened positive for food and/or housing insecurity. The reported rate of food insecurity found in this study does appear somewhat lower than the national average as well as compared to previous studies investigating food insecurity in the pediatric ED, although overall numbers vary widely based on data source.^1^ ^,^ ^22^ ^,^ ^23^ ^,^ ^25^ ^,^ ^27^ We did find higher food insecurity rates than that of the surrounding county in the study year.^28^ Still, our study design was limited by convenience sampling and a 2.8% response rate of participants in the ED, limiting the generalizability of our findings. Other pediatric ED-based studies demonstrate similar challenges, with low response rate (3.6%)^29^ and health-related social need-positive screening rate (16%).^30^ It is important to note that even if families screen positive for social risk, a substantial proportion may still decline assistance.^31^ Similarly, social needs navigation follow-up has been shown to be challenging in the ED setting, with low participation rate (7%)^32^ and persistence of social need (56%) despite participation in navigation services.^19^

In our study, follow-up survey data revealed an overall reduction in the reported rates of food and housing insecurity, yet community resource referral uptake was low. This likely reflects the complexity and burden of patient social circumstances and a multitude of environmental factors. Among those who were food insecure at baseline, almost half no longer screened positive for food or housing insecurity, and among those who were housing unstable at baseline, over 40% no longer screened positive for food or housing insecurity. Those with food and housing insecurity at baseline demonstrated the least reduction in social need, with just under 10% no longer screening positive for either food or housing insecurity at follow-up. However, despite these apparent positive shifts, it is difficult to ascertain whether these developments were associated with ED interventions. Indeed, it seems unlikely given that the majority of caregivers with whom we followed up did not endorse resource use.

It is beyond the scope of this study to discern the etiology of this trend. It is possible that caregivers under-reported resource use, that completion of the survey itself may have precipitated a change, random chance, or a range of other explanations including an interplay of social determinants of health. Interestingly, Kanak et al demonstrated somewhat similar findings using an intervention available on tablet and personal smartphone (the HelpSteps app), reporting that only 23% of caregivers described using the tool.^19^ Only 14% contacted at least one referral agency, yet 44% reported their primary need either completely or somewhat resolved.^19^ As in the current study, Kanak et al found housing needs to be more persistent than food insecurity as well.^19^ Liberman et al also examined social needs interventions in the pediatric ED with trained navigators and noted much greater use of resources (45.6% of those who followed up reported contacting at least one resource); however, it was difficult to determine whether these were housing- and food-related resources as these represented only 21% and 20%, respectively, of referral resources provided.^21^

In the current study, the most common barrier to resource use reported by caregivers was that they either lost or did not receive the referral. While this initially appears somewhat discouraging it also may potentially prove the simplest obstacle to address in future work. It may be helpful to provide electronic forms of resources in addition to written copies, as suggested by caregivers in other pediatric ED-based studies.^21^ ^,^ ^29^ It is remarkable that among caregivers in a study by Cullen et al who screened positive for food insecurity and opted to receive a direct phone call from a food resource agency, only 35.9% were able to be reached, and of those, 31% were no longer interested in food-resource referrals.^23^ It is possible that future research may also elicit appropriate methods of needs reassessment and timing for such reassessment. Increased engagement and collaboration with the community, both with those in need and with those providing resources (ie, food banks), may pave the way for improved screening design and resource information deployment, as well as more successful and increased use of interventions.

The current study reinforces associations demonstrated throughout the literature between food/housing insecurity and caregiver/patient physical and mental health.^1^ ^,^ ^4^ ^–^ ^12^ This serves to underscore the importance of attempting to address food and housing insecurity at every opportunity. The association of neighborhood safety and food and housing insecurity, while not unexpected, likely additionally compounds the chronic illnesses such as anxiety, obesity, and asthma also found in the current study to be associated with food and housing insecurity. Notable demographic associations with food and/or housing insecurity included older age; age also appeared to be associated with the transition from positive to negative screens for food and/or housing. Gonzalez et al also found increasing age and public health insurance to be associated with food insecurity; however, unlike in the current study they did not find associations between food insecurity and chronic health conditions.^25^

The association of age and social need is likely multifactorial and may include variables such as reduced resources available for families with older children, and increased monetary requirements of older children possibly represent more deeply entrenched social need. Interestingly, despite previous literature demonstrating an association between food insecurity and increased healthcare utilization such as ED visits, the current study found somewhat equivocal data.^15^ ^,^ ^17^ One ED visit within the past year was more likely to be associated with food and housing insecurity; however, two or more was not. Additionally, the transition from positive to negative screens was also associated with a slightly increased mean number of ED visits within the prior year. It is difficult to hypothesize what may be driving these seemingly discordant results; however, it is possible that it is the unequal interplay of multiple variables; for example, younger children who are also more likely to transition from positive to negative screens are more likely to visit the ED overall.

## LIMITATIONS

There were several limitations inherent to the design of this study, including the use of convenience sampling with data collectors present only during the day and early evening. This sampling technique may not have captured those with particularly challenging social circumstances, underestimating the true rates of food and housing insecurity, while increasing the likelihood of sample bias and presence of confounding factors. Additionally, this study relied upon self-report for identification of food/housing insecurity as well as resource use; therefore, reporting bias may have impacted our results. Although we attempted to design the study in such a way to reduce potential discomfort as much as possible, financial means and social need in general remain sensitive topics, and concerns regarding privacy and stigma may have contributed further to reporter bias. This is especially pertinent as follow-up surveys were conducted over the phone while initial surveys were completed on electronic tablets, possibly contributing to fluctuations in the reporting of food and/or housing insecurity.

Families were contacted by study research personnel, and this mechanism may be less effective than established closed-loop referral mechanisms in which the community-based social service itself is linked directly with the healthcare institution. The follow-up period of three to six weeks may also be somewhat limited, and it is possible that resource use, especially for more complex needs such as housing, may not effect change within this short period. It is also worth considering, for example, that while food banks are essential social resources to address hunger, they are a temporary solution, and do not increase the ability of a caregiver to purchase adequate food. Difficulties in contacting families for follow-up also presented a significant limitation and restricted our ability to evaluate study interventions. During the study period, researchers at the same institution were also conducting a study examining adverse childhood experiences; as part of this concurrent study, social workers may have been consulted for some of these families, potentially altering resource referral distribution for those families. Lastly, because this study took place during the COVID-19 pandemic the resultant increased social needs and rapidly changing economic landscape likely affected our results, possibly reducing the generalizability of this work.

## CONCLUSION

This study suggests that screening and intervention among two common social determinants of health— food and housing insecurity—may be feasible in a pediatric ED setting. At the same time, it illustrates that achieving widespread participation among families may be a significant challenge. Although a significant proportion of caregivers reported a change in food and housing insecurity on follow-up, it is difficult to ascertain what may have contributed to this finding, especially given the limited response rate and reported resource use. Further social needs-intervention research in the pediatric ED setting should be designed to capture larger response rates (including an assessment of social need disclosure in day and overnight periods), while assessing the performance of closed-loop referral and follow-up mechanisms for those families who indicate a desire for assistance.

## Supplementary Information




