# Supplementary material for: Food and Housing Insecurity, Resource Allocation, and Follow-up in a Pediatric Emergency Department
Source: West J Emerg Med. 2025 Jan 15;26(2):326–37. doi: 10.5811/westjem.19435 (PMC11931713; doi:10.5811/westjem.19435)
Supplement: Supplementary file 1 [file wjem-26-326-s001.docx]

| **Supplementary Table 1. Demographics of those with and without completed follow-up surveys** | | | |
| --- | --- | --- | --- |
| **Characteristics** | **Follow-up survey (n=147)** | **No Follow-up survey (n=269)** | ***p*** |
| ***Demographic*** |  |  |  |
| Patient Age, Mean Years (SD) | 7.61 (5.45) | 7.64 (5.41) | 0.96 |
| Patient Male Sex, n (%) | 75 (51.0%) | 139 (51.7%) | 0.92 |
| Patient Ethnicity and Race |  |  | 0.87 |
| Hispanic White, n (%) | 112 (76.2%) | 213 (79.2%) |  |
| White, n (%) | 19 (13.0%) | 28 (10.4%) |  |
| Black, n (%) | 3 (2.0%) | 3 (1.1%) |  |
| Asian, n (%) | 7 (4.8%) | 9 (3.3%) |  |
| Other, n (%) | 6 (4.1%) | 14 (5.2%) |  |
| Missing, n (%) | 0 (0.0%) | 2 (0.7%) |  |
| Patient Health Insurance |  |  | 0.64 |
| Public, n (%) | 135 (91.8%) | 247 (91.8%) |  |
| Private, n (%) | 12 (8.2%) | 20 (7.4%) |  |
| Self-Pay, n (%) | 0 (0.0%) | 2 (0.7%) |  |
| Language Spoken at Home |  |  | <0.001 |
| English, n (%) | 109 (74.1%) | 136 (50.6%) |  |
| Spanish, n (%) | 33 (22.4%) | 125 (46.5%) |  |
| Vietnamese, n (%) | 3 (2.0%) | 3 (1.1%) |  |
| Other, n (%) | 2 (1.4%) | 5 (1.9%) |  |

**Supplementary Information**

***Appendix A. Initial surveys administered to all participating caregivers***

*Demographics:*

- What is your relationship to the child (e.g. mother, father)? *Parent, Grandparent, Sibling, Other (if other, please specify)*

*-* What language do you speak at home? *English, Spanish, Vietnamese, Other (if other, please specify)*

*-* What is your marital status? *Single/ Never Been Married, Married/Living as Married, Separated/ Divorced/ Widowed*

*-* What is the highest grade or level of schooling you completed? *Never attended school or only kindergarten, Grades 1 through 8 (Elementary), Grades 9 through 11 (Some high school), Grade 12 or GED (High school graduate), College 1 year to 3 years (Some college), College 4 years or more (College graduate), Prefer not to Answer*

- Please give your best guess of your total household income. *< $20,000; $20,000-$99; $40,000-$59,999; $60,000-$79,999; $80,000-$99,999; $100,000+, Prefer not to answer*

- Best means of contact: *Call, Text, Email*

- Name of contact for follow up phone calls:

- Primary Phone Number:

- Secondary Phone Number:

- Secondary Phone Number:

- Primary Email:

- Secondary Email:

1. In general, would you say your health is: *Excellent, Very Good, Good, Fair, Poor*

2. In general, would you say your child’s health is: *Excellent, Very Good, Good, Fair, Poor*

3. Has a doctor ever told you that your child has: *Asthma, Obesity, Diabetes, Anxiety, Other Emotional Challenges (getting upset very easily, feeling very sad), Behavioral Difficulties (fighting, hitting, tantrums, stealing, yelling at others)*

4. Within the past 12 months, I worried whether my food would run out before I got money to buy more: *Often true, Sometimes true, Never true, Don't know*

5. Within the past 12 months, the food I bought just didn't last and I didn't have money to get more: *Often true, Sometimes true, Never true, Don't know*

6. In the past 12 months, have you had trouble paying your rent/mortgage/utility bills? *Yes, No, Don’t Know*

7. In the past 12 months, have you been living in stable housing that you: *Own, Rent, Stay at a friend’s home, I do not live in stable housing*

8. How many times have you moved in the last 12 months?

9. Where do you usually take your child when they are sick or you need advice about their health? *Doctor's Office, Clinic/Health, Center/Urgent Care, Emergency Room, Other (if other please specify)*

10. During the past 12 months, how many times did your child visit a hospital emergency room?

11. Do you feel safe in your neighborhood? *Never, Sometimes, Usually, Always, Don't Know*

12. Are you concerned about your child’s safety in your neighborhood? *Never, Sometimes, Usually, Always, Don't Know*

13. How often can you find fresh fruits and vegetables in your neighborhood? *Always, Usually,*

*Sometimes, Never, Don't Know*

14. How easily can you find fast food (McDonalds, Burger King) in your neighborhood? *Always, Usually, Sometimes, Never, Don't Know*

15. Most of the time, I buy my family's groceries including fruits and vegetables from pharmacies (e.g CVS, Walgreens), Dollar stores, and convenience stores (e.g. 7-11): *Strongly Disagree, Disagree, Neither Agree or Disagree, Agree, Strongly Agree*

16. Most of the time, I buy my family's groceries from grocery stores and supermarkets (e.g. Ralph's, Albertsons, Superior Grocers): *Strongly Disagree, Disagree, Neither Agree or Disagree, Agree, Strongly Agree*

***Appendix B. Surveys administered at 3 and 6-weeks to those screening positive for food and/or housing insecurity***

1. In general, would you say your health is: *Excellent, Very Good, Good, Fair, Poor*

2. In general, would you say your child’s health is: *Excellent, Very Good, Good, Fair, Poor*

3. Have you moved or had a change of home address, housing status, or location where you are sleeping since your child’s discharge from the Emergency Department? *Yes, No*

4. How many times did this occur since your child’s discharge from the ED?

5. Did you use your food or housing referral since you child’s discharge from the ED? *Yes, No*

6. Which referral(s) did you use? Food, Housing

7. Which specific food assistance programs/locations have you visited?

8. How many times have you made use of your food assistance program referral since your child’s discharge from the ED?

9. On a scale of 1 to 5, how helpful was this food assistance program referral in reducing your concerns about food security? *1=Not at all helpful, 2=Slightly; Did not have time, 3= Moderately helpful, 4= Very helpful, 5=Extremely helpful*

10. Which specific housing assistance programs/locations have you visited?

11. How many times have you made use of your housing assistance program referral since your child’s discharge from the ED?

12. On a scale of 1 to 5, how helpful as this housing assistance program referral in reducing your concerns about housing security? *1=Not at all helpful, 2=Slightly; Did not have time, 3= Moderately helpful, 4= Very helpful, 5=Extremely helpful*

13. What prevented you from using your referral? *Lost referral/Did not receive the referral, Did not have time, No transportation available, You were or your child was too ill, No resources in current area, Stigma/privacy concern, Programs did not fit your needs, Other*

14. Within the past 3 weeks, I worried whether my food would run out before I got money to buy more: *Often true, Sometimes true, Never true, Don’t know*

15. In the past 3 weeks, have you had trouble paying your rent/mortgage, utility bills? *Yes, No, Don’t know*

16. In the past 3 weeks, have you been living in stable housing that you: *Own, Rent, Stay at a friend’s home, I do not live in stable housing*

17. Do you feel safe in your neighborhood? *Never, Sometimes, Usually, Always, Don’t know*

18. Are you concerned about your child’s safety in your neighborhood? *Never, Sometimes, Usually, Always, Don’t know*

19. How often can you find fresh fruits and vegetables in your neighborhood? *Always, Usually, Sometimes, Never, Don’t know*

20. How easily can you find fast food (McDonalds, Burger King) in your neighborhood? *Always, Usually, Sometimes, Never, Don’t know*

21. Most of the time, I buy my family’s groceries including fruits and vegetables from pharmacies (e.g. CVS, Walgreens), Dollar sores, and convenience stores (e.g. 7-11): *Strongly Disagree, Disagree, Neither Agree or Disagree, Agree, Strongly Agree*

22. Most of the time I but my family’s groceries from grocery stores and supermarkets (e.g. Ralph’s, Albertsons, Superior Grocers): *Strongly Disagree, Disagree, Neither Agree or Disagree, Agree, Strongly Agree*
